# Low-Carbon Construction and Building Materials

**DOI:** 10.3390/ma19091726

**Published:** 2026-04-24

**Authors:** Junfei Zhang

**Affiliations:** School of Civil and Transportation Engineering, Hebei University of Technology, Tianjin 300401, China; junfeizhang@hebut.edu.cn

## 1. Introduction and Scope

The global construction industry is undergoing an unprecedented transformation toward carbon neutrality, as the production of traditional Portland cement and concrete contributes approximately 8% of global anthropogenic CO_2_ emissions, placing enormous pressure on climate governance and resource conservation [1]. With the continuous expansion of infrastructure construction in emerging economies, the excessive consumption of natural minerals, river sand, and other non-renewable raw materials has further intensified the contradiction between development and sustainability [2]. In this context, low-carbon construction and building materials have become the core driving force for the green upgrading of the construction industry, covering a series of innovative directions such as low-carbon cementitious materials [3], recycled aggregate concrete [4], solid waste resource utilization [5], bio-based building materials [6], functional composite materials [7], and 3D-printed construction materials [8].

In recent years, research into low-carbon building materials has produced fruitful results: the substitution rate of industrial solid waste for cement has continuously improved [9], alkali-activated materials with zero-cement characteristics have developed rapidly [10], green concrete with excellent durability has been gradually applied in engineering [11], and the combination of intelligent technology and material design has opened up new research paradigms [12]. However, there are still prominent bottlenecks restricting the large-scale industrialization of low-carbon building materials. Firstly, the long-term service performance of low-carbon materials prepared from solid waste under complex environments such as freeze–thaw cycles, chloride ion erosion, and high-temperature steaming is not fully clarified, and the degradation mechanism and life prediction model need to be further improved. Secondly, the gap between laboratory basic research and engineering application is clear, and the preparation process of most low-carbon materials is difficult to adapt to large-scale industrial production. Thirdly, the synergistic reaction mechanism of multi-source solid waste in cementitious systems is complex and unclear, which limits the efficient and high-value utilization of various industrial by-products. Fourthly, the integration of low-carbon materials with functional design (such as electromagnetic shielding, thermal insulation, and intelligent sensing) is insufficient, and the multi-performance collaborative optimization technology is immature. Finally, the standard system, carbon accounting method, and market-oriented promotion mechanism of low-carbon building materials are not perfect, leading to slow industrial transformation.

This Special Issue, “Low-Carbon Construction and Building Materials”, aims to gather the latest cutting-edge research results in the field; focus on the physical and chemical mechanisms, mechanical properties, long-term durability, and engineering application of low-carbon building materials; bridge the gap between basic research and practical application; and provide theoretical support and technical solutions for the sustainable development of the construction industry. The collection brings together original research papers and review articles covering green concrete innovation, solid waste recycling, alkali-activated material design, functional building composites, and precast concrete low-carbon technology, fully demonstrating the interdisciplinary and application-oriented characteristics of low-carbon building material research.

## 2. An Overview of the Published Articles

This Special Issue has successfully published six high-quality academic papers, discussing the utilization of coal mine solid waste, municipal solid waste incineration bottom ash, reservoir sediment and other renewable resources; design and performance optimization of alkali-activated materials; development of cement-based electromagnetic shielding composites; intelligent design of materials assisted by machine learning; and comprehensive application of industrial by-products in precast concrete. These studies cover the whole chain of material preparation, mechanism analysis, performance optimization, and engineering application and have both important academic value and significance for engineering guidance.

The first work, by Zhang et al., innovatively applies machine learning to the elemental design of solid waste-based alkali-activated materials [13]. Based on 616 uniaxial compressive strength data of fly ash and granulated blast furnace slag-based alkali-activated materials, the authors trained four tree-based machine learning models, among which the Gradient Boosting Regression (GBR) model had the highest prediction accuracy (R = 0.970; RMSE = 4.110 MPa). SHAP analysis shows that water content and curing age are the most important factors affecting the strength of alkali-activated materials, and the optimal ratio of calcium, silicon, sodium, and aluminum is proposed. This research realizes the intelligent and efficient design of low-carbon cementitious materials and provides a new method for accelerating the research and development of solid waste-based building materials.

The second paper, a critical review by Shao et al., systematically summarizes the comprehensive utilization of industrial by-products in steam-cured precast concrete [14]. In the review, the authors analyze the four stages of the steam curing system (pre-curing, heating, constant temperature, and cooling) and clarify the influence of solid waste on the deformation, pore structure, chloride ion permeability, delayed ettringite formation, and compressive strength of precast concrete. They point out that the incorporation of solid waste can reduce the demolding strength of precast concrete but significantly improve the late strength, optimize the pore structure, and inhibit delayed ettringite formation. In addition, the synergistic effect of multi-source solid waste under high-temperature steam curing is affected by temperature and water–binder ratio. This review provides a comprehensive theoretical reference for the low-carbon production of precast concrete and the efficient utilization of industrial by-products.

The third paper, by Jang et al., [15] develops cement-based electromagnetic interference (EMI) shielding composites by adding carbon nanotubes (CNTs), carbon fibers (CFs), and graphene nanoplatelets (GNPs). Different from previous studies that only focused on total shielding effectiveness, this study focuses on the balance between reflection and absorption losses. The experimental results show that the 50 mm thick composite has a shielding effectiveness of more than 50 dB, and the size and shape of conductive fillers significantly affect the shielding mechanism: fiber-shaped fillers (CNTs and CFs) are conducive to improving the absorption performance, while platelet-shaped GNPs enhance the surface reflection effect. This research provides an important design basis for the development of functional low-carbon building materials with electromagnetic shielding requirements in intelligent buildings.

In the fourth article, Masłoń et al. use water reservoir sediment, concrete dust, and municipal solid waste incineration fly ash to prepare lightweight artificial aggregates and evaluate its physical, mechanical, and ecological properties [16]. The research results show that the prepared artificial aggregate has comparable technological properties to commercial recycled aggregates and can be used for the production of lightweight concrete, which is in line with the concept of circular economy. However, the high leaching rate of chloride and sulfate in the aggregate is still a key problem to be solved, and thermal hardening and autoclaving treatment cannot fully immobilize harmful substances. In this study, the authors point out the direction for improving the ecological safety optimization of waste-based artificial aggregates.

In the fifth contribution, by Tamošaitis et al., the authors take municipal solid waste incineration bottom ash (MSWI) as the raw material and develop alkali-activated concrete by vibro-press forming with an extremely low water-to-solid ratio (W/S < 0.08) [17]. Aiming at the problem of hydrogen gas release caused by metallic aluminum in bottom ash during traditional alkali activation, the study adopts a low water–cement ratio and uses sodium silicate and sodium hydroxide solution to provide the water required for the reaction, combined with metakaolin waste to adjust the ratio of silicon, aluminum, and sodium. The prepared concrete has a compressive strength of up to 40.0 MPa, a tensile strength of 5.60 MPa, and a density of 1950 kg/m^3^, which is equivalent to the performance of vibro-pressed cement paving tiles. This research provides a new technical path for the high-value utilization of municipal solid waste incineration bottom ash in construction products.

The authors of the sixth paper, Sun et al., focus on the high-value utilization of coal mine solid waste and develop a high-porosity cement foam filling material [18]. They systematically study the effects of water–cement ratio, foaming agent dilution ratio, foam stabilizer content, gypsum content, fly ash content, gangue content, and gangue particle size on the mechanical properties, transportation characteristics, and microstructure of the filling material through laboratory tests. The results show that when the fly ash content is less than 30%, the gangue content is less than 30%, and the gangue particle size is less than 0.6 mm, the expansion ratio of the filling material is about three times larger, with good mechanical properties and pumpability. Field tests prove that the material has an excellent control effect on goaf surrounding rock, realizing the safe mining of coal mines and the large-scale disposal of solid waste, which has important economic and environmental benefits.

## 3. Conclusions

This Special Issue, “Low-Carbon Construction and Building Materials”, brings together six high-quality contributions that advance the frontiers of low-carbon construction materials, spanning solid waste valorization, high-performance low-carbon binders, functional composite design, data-driven material development, and low-carbon technologies for precast concrete. Collectively, these works address critical gaps in the understanding of low-carbon material mechanisms, performance optimization, and engineering applicability, offering actionable insights and technical pathways for reducing the carbon footprint of the built environment while meeting structural and functional performance demands. The strong academic attention gained by this collection further underscores the timeliness and relevance of these topics in the global push for sustainable construction.

Looking ahead, the journey toward fully decarbonized construction materials continues to demand sustained innovation and cross-disciplinary collaboration. Further efforts are needed to unravel the long-term durability and degradation mechanisms of low-carbon materials under coupled environmental stresses, enabling accurate service-life prediction and reliable engineering deployment. Scaling up laboratory-proven low-carbon material systems to industrial production requires tailored processing technologies, consistent quality control, and supply-chain solutions that bridge research and real-world implementation. A deeper understanding of synergistic interactions among multi-source solid wastes will unlock higher-volume utilization of industrial by-products, enhancing both material performance and circularity.

Integrating multifunctionality—such as electromagnetic shielding, thermal insulation, and smart sensing—into low-carbon material design will open new avenues for next-generation green and intelligent buildings. Parallel advances in standardized testing methods, carbon accounting frameworks, and carbon labeling systems are essential to create a supportive policy and market ecosystem for low-carbon construction materials. Moreover, the continued fusion of materials science with artificial intelligence, nanotechnology, and digital manufacturing will drive efficient, precise, and sustainable material development, reshaping the research paradigm of these materials.

This Special Issue serves as a milestone in the ongoing evolution of low-carbon construction and building materials, and it is our hope that it will stimulate further research, industrial collaboration, and technological translation toward a low-carbon, circular, and sustainable global construction sector.
